# Generation of a symmetrical trispecific NK cell engager based on a two-in-one antibody

**DOI:** 10.3389/fimmu.2023.1170042

**Published:** 2023-04-04

**Authors:** Julia Harwardt, Stefania C. Carrara, Jan P. Bogen, Katrin Schoenfeld, Julius Grzeschik, Björn Hock, Harald Kolmar

**Affiliations:** ^1^ Institute for Organic Chemistry and Biochemistry, Technical University of Darmstadt, Darmstadt, Germany; ^2^ Biologics Technology and Development, Ferring Darmstadt Laboratory, Darmstadt, Germany; ^3^ Biologics Technology and Development, Ferring Biologics Innovation Centre, Epalinges, Switzerland; ^4^ Centre for Synthetic Biology, Technical University of Darmstadt, Darmstadt, Germany

**Keywords:** trispecific antibody, two-in-one antibody, NK cell engager, common light chain, yeast surface display

## Abstract

To construct a trispecific IgG-like antibody at least three different binding moieties need to be combined, which results in a complex architecture and challenging production of these molecules. Here we report for the first time the construction of trispecific natural killer cell engagers based on a previously reported two-in-one antibody combined with a novel anti-CD16a common light chain module identified by yeast surface display (YSD) screening of chicken-derived immune libraries. The resulting antibodies simultaneously target epidermal growth factor receptor (EGFR), programmed death-ligand 1 (PD-L1) and CD16a with two Fab fragments, resulting in specific cellular binding properties on EGFR/PD-L1 double positive tumor cells and a potent ADCC effect. This study paves the way for further development of multispecific therapeutic antibodies derived from avian immunization with desired target combinations, valencies, molecular symmetries and architectures.

## Introduction

While monoclonal antibodies (mAbs) have tremendous potential for treating a variety of diseases, certain modes of action require two different cells to be positioned in close proximity to achieve the desired therapeutic effect. Due to their monospecificity, this is not possible with a mAb or combination therapy, but can be achieved by combining antibody-like fragments in one molecule (1, 2). Since the first proposition of bispecific antibodies (bsAbs) targeting two independent epitopes in the 1960s (3), they have been extensively explored in translational and clinical studies (4, 5).

One possible mode of action for bsAbs, which are referred to as NK cell engager (NKCE), is the recruitment of natural killer (NK) cells by simultaneously binding to a tumor-associated antigen (TAA) and a specific marker on the surface of NK cells to harness the immune function of NK cells in tumor therapy (6).

Among many other proteins, NK cells express CD16a, also known as FcγRIIIa, which is targeted with low affinity by the Fc region of TAA-bound IgG antibodies (7). These IgG molecules can thus mediate antibody-dependent cellular cytotoxicity (ADCC), an effective mechanism for tumor cell killing by NK cells (8). The anti-CD20 antibody rituximab was the first cytotoxic ADCC-capable mAb to be approved for the treatment of non-Hodgkin´s lymphoma in 1997 (9). Since then, more than 30 cytotoxic antibodies have been developed (10) and considerable efforts have been made to increase the efficacy of these therapeutic antibodies by Fc engineering (11). Preclinical models and clinical outcome of patients have shown that ADCC is one of the most important mechanisms contributing to the therapeutic effect of many approved antibodies, including rituximab, cetuximab and trastuzumab (12). Thereby, binding affinity to CD16a seems to be an important component. The 158V isoform of CD16a, which mediates a stronger binding to IgG1 Fcs compared to its 158F counterpart, has been shown to be positively associated with clinical outcome in patients (13, 14).

To further increase affinity and cytotoxicity, antibody-like molecules have been developed that target CD16a with higher affinity than the wild-type Fc of an IgG1 antibody (15). An example of this are bispecific killer cell engagers (BiKEs) targeting CD16a with one binding arm and a TAA with the other e.g. Epithelial Cell Adhesion Molecule (EpCAM) (16) or CD133 (17), respectively. Trispecific killer engagers (TriKEs) are an improved version of BiKEs with CD16a and TAA targeting single chain variable fragments (scFvs) cross-linked with a human IL-15 moiety, having an additional stimulatory effect on NK cell proliferation and activation (18). Innate Pharma is developing trifunctional NKCEs consisting of antibody fragments targeting two NK cell-activating receptors, CD16a and NKp46, and one TAA including CD19, CD20 and EGFR (19). Some approaches utilize two CD16a engaging binding moieties. The company Affimed has introduced its bispecific ROCK^®^ (Redirected Optimized Cell Killing) antibody platform into the clinic. This platform is based on a tetravalent bispecific antibody consisting of altogether four diabodies with two fragment variable (Fv) domains against CD16a and a TAA (20).

One TAA that has already shown impressive outcomes *in vitro* and *in vivo* in combination with CD16a as an NKCE (19) and has even been clinically validated in phase 1/2a clinical trials (AFM24, NCT 04259450) as part of the bispecific ROCK^®^ platform (20) is epidermal growth factor receptor (EGFR). Overexpression of EGFR has been reported in a variety of cancers, where it is involved in tumor progression and metastasis (21–23).

Furthermore, AFM24 is also being investigated in phase 1/2a clinical trials in combination with atezolizumab, an anti-programmed death-ligand 1 (PD-L1) antibody (NCT05109442). PD-L1 is overexpressed in many malignancies and represents a mechanism by which cancer evades immune surveillance (24, 25). Moreover, NK cell activity can be negatively affected by immune checkpoints such as PD-1/PD-L1 axis (26). Therefore, the combination of targeting CD16a, EGFR and PD-L1 could provide significant clinical benefit also in view of the recently reported finding that tumor-specificity can be elevated by simultaneous targeting EGFR and PD-L1 by a bispecific antibody (27).

Recently, we reported the isolation of a chicken-derived two-in-one antibody (HCP-LCE) targeting EGFR and PD-L1 with two independent paratopes on a single Fab (28). Unlike classical IgG-like bispecific antibodies that require heterodimerization of the heavy chains and correct light chain pairing (29), two-in-one antibodies are symmetrical molecules consisting of two identical heavy and light chains (30). Consequently, they can be produced without engineering of constant chains, eliminating the need to incorporate unnaturally occurring amino acids as found in knob-into-hole antibodies (31) or orthogonal Fab interfaces (32). This chicken-derived two-in-one antibody inhibits EGFR signaling by binding to dimerization domain II and blocks the PD-1/PD-L1 interaction (28). Notably, while individual affinities to EGFR and PD-L1 are moderate with K_D_ values in the triple and double digit nanomolar range, the two-in-one combined antibody displays high affinity binding to cells expressing both targets (28).

Although most approved therapeutic antibodies have been generated using rodent immunization (33), immunization of chickens has gained interest in the scientific community. Due to the wider phylogenetic distance from humans, chicken immunization may result in antibodies targeting epitopes not accessible upon immunization of rodents (34, 35). In addition, gene diversification in birds allows library generation with a single set of primers, significantly reducing hands-on time and costs compared to rodents (36). Subsequently, chicken-derived antibodies with high affinity can be isolated using yeast surface display (YSD) in combination with fluorescence-activated cell sorting (FACS) (36–38).

In this study, we describe the construction and characterization of a symmetric trispecific common light chain antibody based on a previously identified two-in-one antibody targeting EGFR and PD-L1. By utilization of the light chain of this two-in-one antibody as a common light chain (cLC) in a chicken-derived anti-CD16a immune heavy chain library, a novel CD16a engaging cLC antibody was identified and subsequently fused in a head-to-tail setup with the parental two-in-one antibody. The resulting trispecific antibody (HC16-HCP) has the ability to simultaneously bind PD-L1, EGFR and CD16a with six independent paratopes on four single Fabs, without additional engineering of the CDR regions and in a generic, symmetrical architecture. Comparable to the two-in-one antibody HCP-LCE, it exhibits specific cellular binding on EGFR and PD-L1 double positive tumor cells, blocks the PD-1/PD-L1 axis and mediates a potent ADCC effect as an NKCE. This work paves the way for the generation of chicken-derived trispecific common light chain immune cell engager molecules in a straightforward manner and facilitates subsequent process development through its symmetrical architecture.

## Results

### Library generation and screening

**Figure 1:**
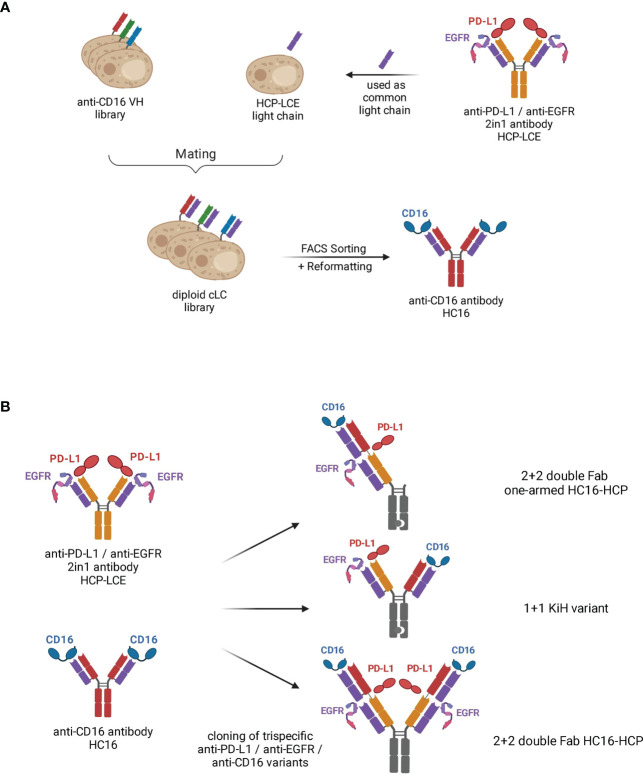
Schematic representation demonstrating the generation of trispecific antibody variants based on the two-in-one antibody HCP-LCE. **(A)** The VL fragment of the anti-PD-L1/anti-EGFR two-in-one antibody HCP-LCE was paired with an anti-CD16a VH yeast surface display library by yeast mating. FACS screening and subsequent reformatting into full-length antibody format enabled the isolation of the anti-CD16 antibody HC16. **(B)** Based on the antibodies HCP-LCE and HC16, three different trispecific anti-PD-L1/anti-EGFR/anti-CD16 antibody variants were generated. Created with BioRender.com.

Recently, we isolated a chicken-derived two-in-one antibody (HCP-LCE) that simultaneously targets EGFR and PD-L1 with the same Fv region (28). Notably, additional studies revealed that the two-in-one antibody targets keratinocytes and fibroblasts with a lower on-cell affinity than cetuximab (**Supplementary Figures 1A, B**). Therefore, HCP-LCE may be expected to cause milder side effects compared to EGFR high affinity binding antibodies such as cetuximab, for which the main side effect is skin toxicity, including rash, dry skin, hair growth disturbances and nail changes (39, 40). Based on its ability to inhibit EGFR signaling by binding to dimerization domain II, block the PD-1/PD-L1 interaction and mediate specific cellular recognition to EGFR/PD-L1 double-positive tumor cells, HCP-LCE was chosen as a starting molecule to introduce a NK cell engaging module. Since the most straightforward approach to circumvent light chain pairing problems in multispecific antibodies is the use of a common light chain that pairs with all VH moieties (41, 42), the HCP-LCE light chain was combined with a VH library from chickens immunized with the CD16a extracellular domain aimed at screening for high affinity binders (**Figure 1**). To this end, the heavy chain yeast surface display library was generated by amplification of VH genes from cDNA derived from a chicken immunized with CD16a-Fc and boosted with monomeric CD16a and subsequently inserted into a pYD_1_-derived vector encoding a human CH_1_ domain by homologous recombination in EBY100 yeast, as previously described (43). The heavy chain diversity was combined with BJ5464 yeast cells encoding the HCP-LCE VL-CLλ fragment by yeast mating (**Figure 1**), resulting in an estimated library size of 4x10^8^ variants.

The diploid common light chain yeast library was screened by FACS over four consecutive sorting rounds using His_6_-tagged monomeric CD16a, resulting in an enrichment of a binding population (**Supplementary Figure 2A**). Sequence analysis of eight randomly chosen clones revealed one distinct VH sequence, which was enriched during the sorting process. The isolated anti-CD16a antibody is referred to as HC16. Binding to the His_6_-Tag of the protein could be excluded by binding analysis of a yeast single clone to an unrelated His_6_-tagged protein (**Supplementary Figure 2B**).

### Cloning and biophysical characterization of trispecific antibodies

For the generation of EGFR, PD-L1 and CD16a targeting antibody variants, Fab fragments of the two-in-one antibody HCP-LCE and the anti-CD16a antibody HC16 were fused in a head-to-tail setup into a 2 + 2 double Fab format (termed HC16-HCP) and an 1 + 1 IgG-like format (termed KiH variant) *via* Golden Gate Cloning. For the symmetrical 2 + 2 variant HC16-HCP, similar to a Fabs-in-tandem immunoglobulin (FIT-Ig) (44), two Fabs were located on each heavy chain, connected by a flexible linker. Contrary to the crisscross orientation of the FIT-Igs (44), the VH of the inner Fab was connected to the CH_1_ domain of the outer Fab. Since the light chains were identical for each binding moiety of the 2 + 2 variant HC16-HCP, the possibility of mismatch between heavy and light chains was eliminated. The Fc exhibited the LALA mutation to circumvent Fc : CD16a-interactions (45, 46). Following production in Expi293F cells, HC16-HCP was purified like a conventional IgG *via* Protein A affinity chromatography due to its symmetrical architecture, circumventing the need to include unnaturally occurring amino acid sequences in constant chains as found in knob-into-hole antibodies or orthogonal Fab interfaces (31). In addition, a trispecific 1 + 1 variant (KiH variant; **Figure 1**) and a one-armed double Fab HC16-HCP variant (termed oaHC16-HCP) were generated in which the Fc fragments exhibited the knob-into-hole mutations to force heterodimerization of the heavy chains (31). To ensure the isolation of heterodimers only, a TwinStrepII-Tag was fused to the C-terminus of the Knob heavy chain (HC), whereas a His_6_-Tag was placed C-terminally to the Hole-HC, enabling two-step purification *via* IMAC followed by StrepTactin purification as previously described (28).

SDS-PAGE analysis revealed the presence of all expected heavy and light chains under reducing conditions, as well as the expected molecular size under non-reducing conditions without degradation products (**Supplementary Figure 3A**). For the KiH variant, the two heavy chains run higher than the unmodified two-in-one HCP-LCE heavy chains, due to the additional Twin-StrepII- and His_6_-tag used for purification. The significantly higher molecular weight of the 2 + 2 variant HC16-HCP compared to a full-length antibody was confirmed during size-exclusion chromatography (SEC) by the lower retention time. SEC profiles demonstrated that HC16-HCP exhibited favorable properties with almost no measurable aggregation (**Supplementary Figure 3B**). Thermostability investigated by SYPRO Orange revealed melting temperatures between 64.0°C and 66.0°C, with the parental antibodies HCP-LCE and HC16 exhibiting 58.9°C and 61.5°C respectively, indicating no reduction in thermal stability (**Table 1**).

**Table 1:** Biophysical properties of HCP-LCE, HC16-HCP, oaHC16-HCP, KiH including affinity, kinetic binding rates and melting temperatures.

Antibody	K_D_ [nM]			k_on_ [M^-1^ s^-1^]			k_dis_ [s^-1^]			T_M_ [°C]
	EGFR	PD-L1	CD16a	EGFR	PD-L1	CD16a	EGFR	PD-L1	CD16a	
HC16-HCP	331 ± 29.9	68.0 ± 1.82	17 ± 0.21	5.06 x 10^5^ ± 3.39 x 10^4^	7.75 x 10^5^ ± 1.58 x 10^4^	1.37 x 10^5^ ± 1.14 x 10^3^	1.68 x 10^-1^ ±	5.26 x 10^-2^ ± 9.16 x 10^-4^	2.33 x 10^-3^ ± 2.14 x 10^-5^	66.0°C
oaHC16-HCP	463 ± 51.2	64.8 ± 1.84	19.9 ± 2.01	4.22 x 10^5^ ± 3.5 x 10^4^	8.83 x 10^5^ ± 1.90 x 10^4^	1.36 x 10^5^ ± 9.76 x 10^2^	1.96 x 10^-1^ ± 1.43 x 10^-2^	5.73 x 10^-2^ ± 1.06 x 10^-3^	2.70 x 10^-3^ ± 1.91 x 10^-5^	64.0°C
KiH	320 ± 26.8	57.7 ± 4.10	21.9 ± 0.23	5.63 x 10^5^ ± 3.50 x 10^4^	1.17 x 10^6^ ± 6.24 x 10^4^	1.14 x 10^5^ ± 1.07 x 10^3^	1.80 x 10^-1^ ± 1.01 x 10^-2^	6.76 x 10^-2^ ± 3.17 x 10^-3^	3.09 x 10^-3^ ± 2.10 x 10^-5^	64.5°C
HC16	–	–	18.3 ± 0.20	–	–	1.55 x 10^5^ ± 1.23 x 10^3^	–	–	2.83 x 10^-3^ ± 2.14 x 10^-5^	61.5°C
HCP-LCE	236 ± 10.7	78.3 ± 1.36	–	4.15 x 10^5^ ± 1.58 x 10^4^	9.73 x 10^5^ ± 1.37 x 10^4^	–	9.81 x 10^-2^ ± 2.41 x 10^-3^	7.62 x 10^-2^ ± 7.78 x 10^-4^	–	58.9°C

### Affinity measurement

**Figure 2:**
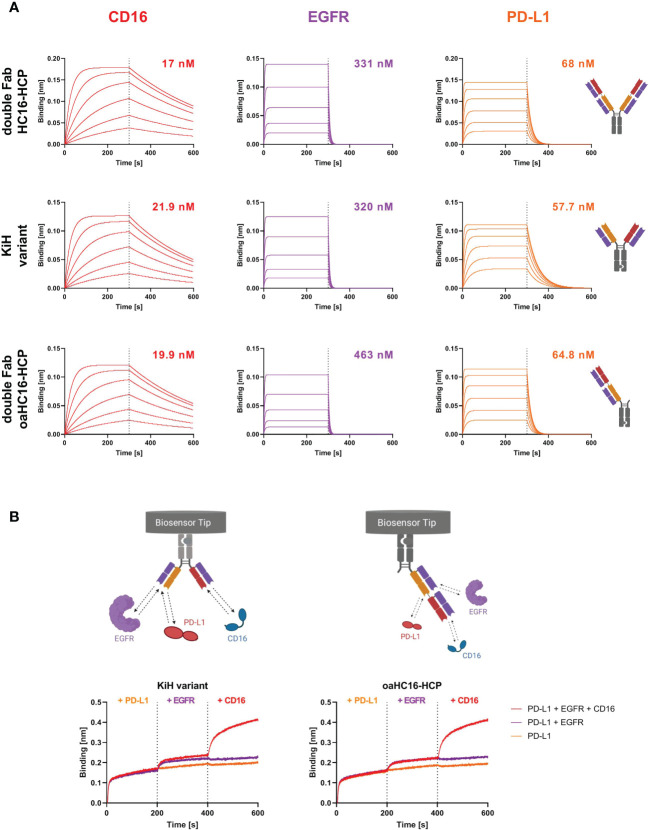
Characterization of antigen binding of the trispecific antibody variants by BLI-measurements. **(A)** BLI-measurements of the double Fab variant HC16-HCP, the knob-into-hole (KiH) variant and the one-armed (oa) double Fab variant oaHC16-HCP against CD16a, EGFR and PD-L1. All antibody variants target the three respective antigens with comparable K_D_ values. **(B)** BLI-assisted simultaneous binding assay. The KiH variant and the oa double Fab variant were loaded onto AHC biosensors and antigens were added stepwise, demonstrating simultaneous PD-L1, EGFR and CD16a binding. Created with BioRender.com.

BLI measurements were performed to determine the affinity of the different trispecific variants to all three targets of interest. All three antibody variants were able to target CD16a as well as EGFR and PD-L1 (**Figure 2A**). The binding affinities to EGFR and PD-L1 were in similar ranges to those previously published for the two-in-one antibody HCP-LCE (28). This suggests that the outer CD16a targeting Fab arm does not significantly interfere with EGFR and PD-L1 binding of the inner two-in-one Fab arm of the 2 + 2 variant HC16-HCP and the oaHC16-HCP variant. The parental antibody HC16 exclusively targeted CD16a with a K_D_ value of 18 nM and exhibited no binding to EGFR (**Supplementary Figure 4**). The binding affinities of the trispecific variants to CD16a were in similar ranges to that of the parental antibody (**Figure 2**). This supports the notion that EGFR binding of the HCP-LCE light chain is supported by the presence of the anti-PD-L1 heavy chain (28).

In order to maintain tumor selectivity of the two-in-one antibody and additionally obtain effector cell recruitment, simultaneous binding to all three targets is essential. To analyze whether CD16a, EGFR and PD-L1 can be targeted simultaneously with two Fab arms, the KiH variant and the double Fab oaHC16-HCP variant were loaded onto anti-human IgG Fc Capture (AHC) biosensors and incubated sequentially with the target proteins of interest. Here, it was important to use the one-armed variant, since the symmetrical 2 + 2 HC16-HCP is hexavalent. Binding to PD-L1 first, EGFR second and CD16a third was considered (**Figure 2B**). Both variants were able to bind all three targets simultaneously with two Fab fragments, supporting the suggestion that the outer Fab arms does not disrupt the EGFR and PD-L1 binding properties of the two-in-one Fab.

### EGF and PD-1 competition

**Figure 3:**
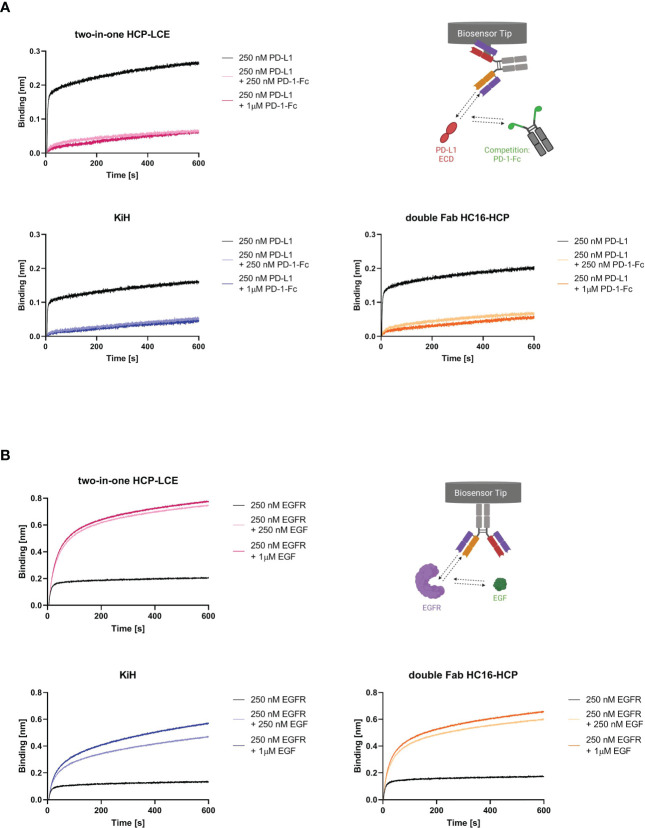
EGF and PD-1 competition assay by BLI. **(A)** BLI-assisted PD-1 competition assay. The two-in-one antibody HCP-LCE (pink), the KiH variant (blue) and the double Fab variant HC16-HCP (orange) were loaded onto FAB2G biosensors and subsequently associated to PD-L1 preincubated with varying PD-1 concentrations. Binding of the antibody variants to PD-L1 at different PD-1 concentrations reveal dose-dependent binding. **(B)** BLI-assisted EGF competition assay. The two-in-one antibody HCP-LCE (pink), the KiH variant (blue) and the double Fab variant HC16-HCP (orange) were loaded onto AHC biosensors and subsequently associated to EGFR preincubated with varying EGF concentrations. The trispecific antibody variants bind to EGFR despite EGF binding. Created with BioRender.com.

To study whether not only the binding kinetics of the two-in-one antibody were preserved but also the antibody-mediated ligand receptor blocking properties, BLI-assisted competition assays were performed. To investigate PD-1/PD-L1 competition, the respective antibodies were loaded onto FAB2G biosensors and associated with 250 nM PD-L1 preincubated with either 250 nM or 1000 nM PD-1-Fc. Binding of both trispecific antibody variants to PD-L1 was significantly impaired in the presence of PD-1 (**Figure 3A**), suggesting that the ability of the two-in-one antibody to target and block the PD-1/PD-L1 interaction site is preserved.

HCP-LCE targets EGFR domain II, which is involved in receptor dimerization (28, 47). Since EGF binds simultaneously to EGFR domains I and III, the antibody does not block the EGF/EGFR interaction. Ligand binding and EGFR dimerization are essential for full EGFR activation, so HCP-LCE inhibits EGFR signaling by inhibiting dimerization of EGFR. For analysis of EGF competition, AHC biosensors were loaded with the respective antibody and associated with 250 nM EGFR preincubated with 250 nM or 1000 nM monomeric EGF. Binding of the antibodies to the EGFR/EGF complex resulted in an increase in layer thickness compared with binding to EGFR alone (**Figure 3B**), indicating that both trispecific antibodies, like the two-in-one antibody, do not target the interaction site of EGF and EGFR.

### Cellular EGFR and PD-L1 binding

**Figure 4:**
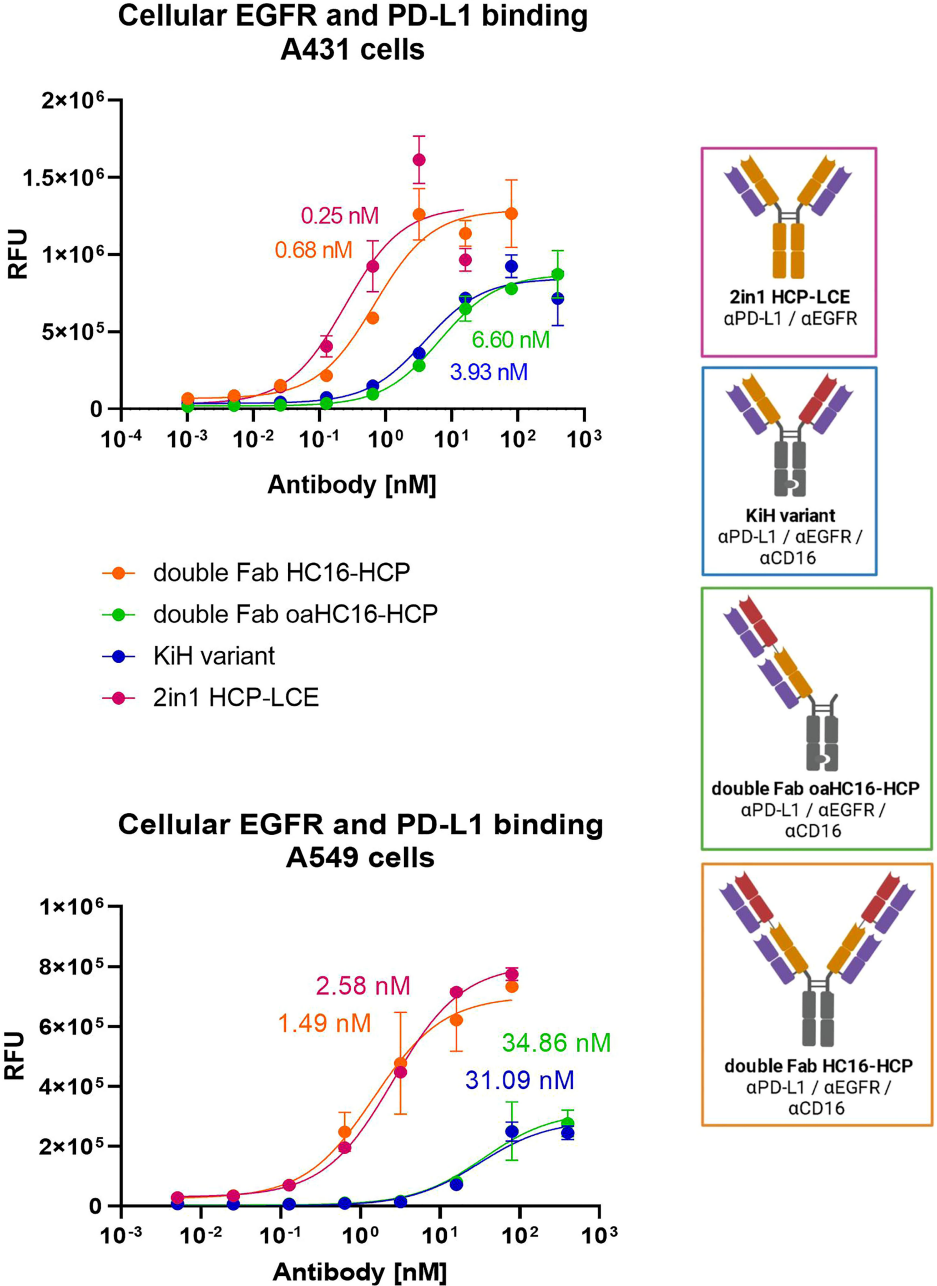
Cellular binding of trispecific variants on EGFR and PD-L1 double positive cells. Cell titration of double Fab HC16-HCP (orange), double Fab oaHC16-HCP (green), KiH variant (blue) and HCP-LCE (pink) on EGFR/PD-L1 double positive A431 and A549 cells. A variable slope three-parameter fit was used to fit the resulting curves. EC50 values of A431 cellular binding: HCP-LCE, 0.25 nM; HC16-HCP, 0.68 nM; KiH, 3.93 nM; oaHC16-HCP, 6.60 nM. EC50 values of A549 cellular binding: HCP-LCE, 2.58 nM; HC16-HCP, 1.49 nM; KiH, 31.09 nM; oaHC16-HCP, 34.86 nM. All measurements were performed in duplicates and the experiments were repeated at least three times, yielding similar results. Created with BioRender.com.

To ensure that the 2 + 2 HC16-HCP variant shows comparable tumor-targeting to the two-in-one antibody HCP-LCE, cellular binding experiments were performed on EGFR/PD-L1 double positive A431 and A549 cells by flow cytometry. Cells were stained with the respective antibodies at a concentration ranging from 0.1 pM to 80 nM for A431 cells and from 5.12 pM to 80 nM for A549 cells utilizing a five-fold dilution series. Binding was verified using an anti-human Fc PE detection antibody. As a control, the cells were stained with the KiH variants at a concentration ranging from 0.1 pM to 400 nM for A431 cells and from 5.12 pM to 400 nM for A549 cells. The 2 + 2 antibody HC16-HCP exhibited cellular. Binding with an EC_50_ value of 0.68 nM on A431 cells and 1.49 nM on A549 cells, which was within the range of the two-in-one antibody (**Figure 4**). The EC_50_ values of the 1 + 1 KiH variant and the oaHC16-HCP were also in a similar range for both cell lines. However, the antibody variants consisting of only one two-in-one Fab exhibited a higher EC_50_ value by a factor of approximately 6 on A431 cells and about 30 on A549 cells (**Figure 4**). Since EGFR expression on A431 cells is significantly higher than that on A549 cells (48), lower EC_50_ values were expected on A431 cells. The similar EC_50_ values of the antibodies HCP-LCE and HC16-HCP, as well as of the 1 + 1 KiH variant and oaHC16-HCP, were also in line with expectations, as they each consist of the same number of EGFR/PD-L1 binding Fab fragments. The different valency of EGFR/PD-L1 binding likely contributes to the lower EC_50_ value of the double Fab HC16-HCP variant, which, contrary to the KiH variants, targets the antigens bivalently instead of monovalently. Similarly, the lower binding maximum observed for the monovalent constructs was to be expected due to the lack of avidity effects. The antibodies did not show binding to EGFR/PD-L1 double negative Daudi cells, excluding non-specific cellular binding (**Supplementary Figure 5**). These data indicate that the tumor-targeting ability of the two-in-one Fab fragment remains functional even when another antibody fragment is fused to it. In addition, tumor selectivity is assumed to be maintained as demonstrated by cellular MRC-5 binding (**Supplementary Figure 1C**).

### Antibody-dependent cell-mediated cytotoxicity reporter assay

**Figure 5:**
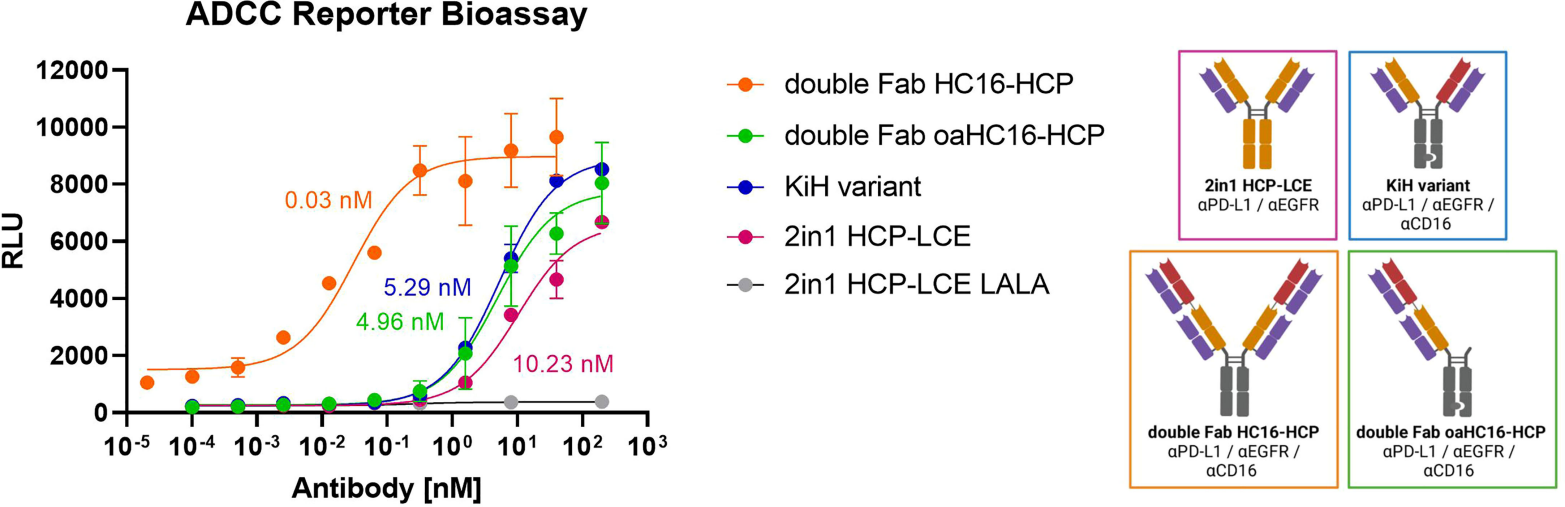
ADCC cell-based reporter assay. The trispecific constructs KiH (blue), HC16-HCP (orange), oaHC16-HCP (green) and HCP-LCE (grey) with the LALA mutation were tested in comparison to HCP-LCE with a wildtype IgG1 Fc (pink). EC_50_ values: HC16-HCP, 0.03 nM; oaHC16-HCP, 4.96 nM; KiH, 5.29 nM; HCP-LCE, 10.23 nM. Luciferase activity is plotted against the logarithmic antibody concentration. A variable slope three-parameter fit was used to fit the resulting curves. All measurements were performed in duplicates and the experiment was repeated at least three times, yielding similar results. Created with BioRender.com.

Fundamental aspects of an NK-cell engager are immune cell stimulation and cell killing. To compare the ADCC effect of the trispecific variants, the Promega ADCC luminescent reporter assay was used with EGFR/PD-L1 double-positive A431 cells as target cells. All variants contained the LALA mutation , except for HCP-LCE were, additional to a LALA variant, an antibody with wild-type Fc was tested. As expected, HCP-LCE exhibiting the LALA mutation failed to mediate an ADCC effect even at high antibody concentrations. The 1 + 1 trispecific KiH variant mediated an ADCC effect that showed a two-fold higher induction than the bivalent EGFR and PD-L1 binding HCP-LCE exhibiting a wild-type IgG1 Fc. The 2 + 2 HC16-HCP trispecific showed the most potent ADCC effect, indicating the effector cell engaging properties of the anti-CD16a Fab fragment (**Figure 5**). No signal was detected in the absence of tumor cells, suggesting that HC16-HCP does not activate NK cells *via* CD16a cross-linking (**Supplementary Figure 6**).

In summary, the generated trispecific antibody of symmetric architecture (HC16-HCP) is able to simultaneously target PD-L1, EGFR and CD16a with six independent paratopes on four individual Fab fragments. Comparable to the two-in-one antibody HCP-LCE, it exhibits specific cellular binding to EGFR and PD-L1 double positive tumor cells, blocks the PD-1/PD-L1 axis and mediates a potent ADCC effect as an NKCE.

## Discussion

Trispecific antibodies are considered promising molecules since, compared to bispecific antibodies, the additional specificity expands the repertoire of targets and provides flexibility in designing the antigen binding valence. Consequently, trispecific antibodies are available in many different formats and target combinations (49–51).

In this study, we generated the first chicken-derived trispecific antibody based on a two-in-one antibody simultaneously targeting EGFR, PD-L1 and CD16a with two Fab fragments. To this end, the light chain of the chicken-derived EGFR and PD-L1 targeting two-in-one antibody HCP-LCE (28) was paired with a chicken-derived anti-CD16a heavy chain immune library by yeast mating. Isolation of the anti-CD16a antibody HC16 tolerating the common light chain was performed by four rounds of FACS-based selection using YSD. Based on the antibodies HCP-LCE and HC16, two different trispecific common light chain antibodies were generated, which differ in their structure and antigen binding valency. One of the two variants consists of a 2 + 2 format (HC16-HCP), while the KiH variant is constructed like a conventional 1 + 1 IgG-like antibody. The tumor-targeting ability of the two-in-one Fab fragment remained intact and the trispecific antibody variants additionally showed a potent ADCC effect.

Most trispecific antibodies consist of three independent monomeric antigen binding units (49, 50). Another trispecific antibody that resembles the structure of the 1 + 1 trispecific variant is the CD38×CD3×CD28 crossover variable domain (COVD) antibody SAR442257. Here, CD38 is targeted by a conventional Fab and the other arm consists of two linked Fv fragments targeting CD3 and CD28 (52). SAR442257 is currently investigated in a phase I clinical trail (NCT04401020). The same molecular architecture was used by Xu et al. to generate an antibody that targets three different epitopes of HIV envelope (53). Nevertheless, the structure of the 1 + 1 trispecific variant resembles that of a natural IgG more closely, since it consists of two conventional Fab fragments. One advantage of a mAb-like structure is that immunogenicity is expected to be like that of a conventional mAb.

A class of antibodies close to the 2 + 2 trispecific HC16-HCP variant include FIT-Ig molecules (44). FIT-Igs are symmetrical and tetravalent IgG-like bispecific molecules, where two Fabs are fused directly in a crisscross orientation leading to correct VH/VL pairing. A similar structure results in similar advantages which include simple purification of homodimers using standard procedures without extensive optimization. FIT-Ig molecules demonstrate favorable drug-like properties, *in vitro* and *in vivo* functions, and efficient manufacturing for commercial development (44, 54), which is also expected for the 2 + 2 trispecific variant.

Remarkably, the inner HCP-LCE arm of the 2 + 2 variant exhibits binding kinetics comparable to the parental two-in-one antibody. Previous studies using 2 + 2 bispecific antibodies found that the affinity of some mAbs at the inner position decreased slightly, whereas no major effects on affinity were observed for other antibodies, which most likely depends on the shape of the paratope of the inner Fab (44, 54–56). This highlights the suitability of two-in-one antibodies for use in a 2 + 2 architecture. Interestingly, the newly isolated antibody HC16 carrying the two-in-one light chain exclusively targets CD16a, corroborating the notion that EGFR binding of the light chain is supported by the presence of the PD-L1 heavy chain (28), while no contribution to binding or even adverse effects are provided by the CD16a binding VH domain.

By utilizing the HCP-LCE Fab arm as tumor targeting fragment, the trispecific molecules of this study might have increased tumor selectivity. Koopmans and coworkers demonstrated elevated tumor specificity by an EGFR×PD-L1 antibody (27). Because of the comparatively low affinity of HCP-LCE to EGFR, increased target expression, which is predominantly found on tumor cells (57), is required for targeting of the antibody. The low binding affinity could cause EGFR binding exclusively on cells that additionally express PD-L1 due to spatial proximity and local concentration, based on the same concept described for bispecific antibodies targeting CD47 (58–60). This notion is supported by the lower on-cell affinity to MRC-5 and NHEK cells compared to cetuximab. The associated increased tumor selectivity would lead to a more favorable safety profile of the trispecific antibodies.

The design of the two reported trispecific antibody variants varies mainly in their valency towards EGFR, PD-L1 and CD16a, with 2 + 2 HC16-HCP binding bivalently and 1 + 1 variant only monovalently. The effect of bivalent target binding was primarily seen in the cellular binding assay on EGFR and PD-L1 double positive cells, which resulted in lower EC_50_ values. In addition, a significant more potent ADCC effect was observed with bivalent CD16a binding.

Bivalent targeting of CD16a by the 2 + 2 variant HC16-HCP might result in NK cell fratricide after crosslinking two NK cells. Preclinical studies with NKCEs and TriKEs have reported low/no NK cell-mediated fratricide in *in vitro* cytotoxicity assays despite the potential co-engagement of CD16a or NKp46/CD16a on separate NK cells (19, 20). Moreover, the use of an effector-competent Fc could induce even stronger NK cell activation. However, NK cell activity also depends on the distance between target and effector cell (61–63). Therefore, an anti-CD16a Fab adjacent to the tumor arm targeting NK cells might mediate a more favorable distance between target and effector cells than an Fc.

**Figure 6:**
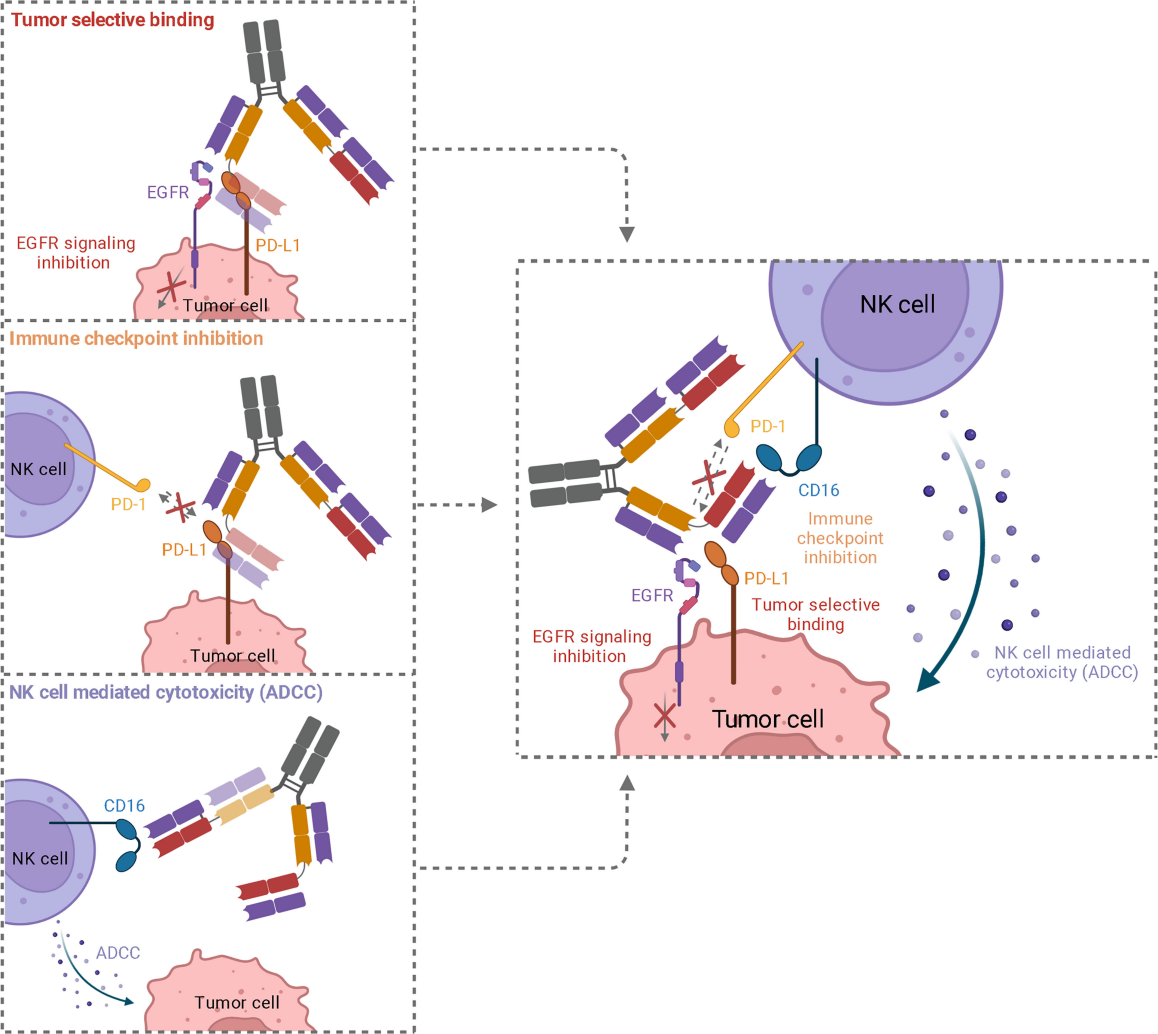
Schematic representation of the three functionalities of trispecific 2 + 2 antibody variant HC16-HCP. While simultaneous binding of EGFR and PD-L1 mediated an elevated tumor specificity, EGFR downstream signaling is inhibited and PD-L1 is blocked, inhibiting interaction with PD-1 on NK cells. The recruitment of cytotoxic NK cells *via* CD16a engagement leads to an effective ADCC.

In summary, the trifunctional antibodies exhibit the following properties: i) dual tumor-targeting by EGFR and PD-L1 binding to increase tumor selectivity, ii) immune checkpoint inhibition by blocking the PD-1/PD-L1 axis, and iii) potent NK cell-mediated cytotoxicity by CD16a targeting (**Figure 6**). As a result, the symmetrical 2 + 2 antibody mediates tetravalent TAA binding and bivalent NK cell binding. To our knowledge, this is the first hexavalent antibody of this format. The advantages of the symmetrical 2 + 2 and the IgG-like 1 + 1 trispecific constructs may pave the way to generate more trispecific molecules based on a two-in-one antibody. In this context, different target combinations and modes of action are possible.

## Material and methods

### Plasmids and yeast strains

For yeast surface display, pYD_1_-derived vectors (Yeast Display Vector Kit, version D, #V835-01, Thermo Fisher Scientific) were used. The heavy chain encoding plasmid contained the *AGA2* signal peptide, followed by the VH-CH_1_ sequences and the *AGA2* gene, a tryptophan auxotrophic marker and an ampicillin resistance. The light chain plasmid encoded an αMFpp8 signal sequence, followed by the HCP-LCE (28) VL-CLλ sequence, a leucine auxotrophic marker as well as a kanamycin resistance gene. Plasmid gene expression was controlled by the galactose-inducible promotor (*GAL_1_*). For soluble expression of full-length and double Fab chimeric antibodies, pTT5-derived vectors (64) encoding either the heavy or light chain constant domain were utilized. The KiH variant was expressed using pTT5-derived vectors encoding the full-length chimeric antibody with either a knob or hole mutation (31) within the CH_3_ sequence and a C-terminal His_6_- or Twin-StrepII-Tag, respectively. For the one-armed double Fab variant, a pTT5-derived vector encoding the Hinge CH_2_-CH_3_ domain with a hole mutation and a C-terminal Twin-StrepII-Tag was used.

For Fab display, Saccharomyces cerevisiae strains EBY100 [MATa URA3-52 trp1 leu2Δ1 his3Δ200 pep4::HIS3 prb1Δ1.6R can1 GAL (pIU211:URA3)] (Thermo Fisher Scientific) and BJ5464 (MATα URA3-52 trp1 leu2Δ1his3Δ200 pep4::HIS3 prb1Δ1. 6R can1 GAL) (American Type Culture Collection) were transformed with the plasmids containing the heavy and light chain genes, respectively. Cultivation of haploid and diploid yeasts in YPD, SD-CAA and SG-CAA media was performed as previously described (65).

### Library generation and sorting

For yeast library generation, the VL-CLλ fragment of the two-in-one antibody HCP-LCE (28) was combined with a chicken-derived anti-CD16a VH-CH_1_ library (43). The HCP-LCE VL gene was amplified by PCR using Q5 polymerase (NEB) and the light chain pYD_1_ vector was linearized utilizing BamHI-HF (NEB) and NheI-HF (NEB) according to the manufacturer´s protocol. Homologous recombination of HCP-LCE VL gene in pYD_1_ was performed in BJ5464 yeast cells according to the protocol described by Bernatuil et al. (65). In order to combine the anti-CD16a heavy chain diversity with the common HCP-LCE light chain for subsequent Fab display, yeast mating was performed as described previously (66).

For library sorting, the diploid yeast library was grown overnight in SD-Trp-Leu medium at 30°C and 120 rpm. The next day, cells were harvested by centrifugation and used to inoculate SG-Trp-Leu medium at an OD_600_ of 1.0. After incubation at 30°C and 120 rpm overnight, cells were harvested by centrifugation, washed once with PBS-B [PBS + 0.1% (w/v) BSA], and incubated on ice with 1µM or 200nM biotinylated CD16a-His_6_ (produced in-house) or CD16a-His_6_ (produced in house) for 30 minutes. Following a PBS-B wash, cells were incubated with a goat anti-human-Lambda Alexa Fluor 647 F(ab’)2 antibody (SouthernBiotech, diluted 1:75) to detect Fab surface presentation and Streptavdin-APC conjugate (Thermo Fisher Scientific, diluted 1:75) or a 6x-His Tag antibody (Fisher Scientific, diluted 1:75) to detect target binding for 15 minutes on ice. An additional PBS-B was step was followed by FACS screening of the cells using a Sony SH800S.

### Reformatting, expression and purification of full-length, one-armed and trispecific antibodies

Plasmid isolation from yeast cells was performed using the Zymoprep Yeast Plasmid Miniprep Kit (Zymo Reasearch) according to the manufacturer’s protocol. The isolated plasmids were transformed into *E. coli* XL1-Blue and sequenced at Microsynth Seqlab (Göttingen). The resulting VH gene was amplified by PCR using Q5 polymerase (NEB) according to the manufacturer’s protocol, incorporating *SapI* sites for subsequent Golden Gate cloning into pTT5-derived vectors as previously described (43). Expi293F cells (Thermo Fisher Scientific, A14527) were transiently transfected with Expifectamine293 (Thermo Fisher Scientific) for soluble expression following the manufacturer’s instructions. The cells were cultured in Expi293 Expression medium (Thermo Fisher Scientific) at 37°C and 8.0% CO_2_ at 110 rpm. For purification of full-length and double Fab antibodies, sterile-filtered cell culture supernatant was applied to a Protein A HP column (GE Healthcare) five days after transfection using an ÄKTA pure system (GE Healthcare). The KiH and one-armed variant were captured by IMAC (HisTrap HP, GE Healthcare), followed by Strep-Tactin XT affinity chromatography according to the manufacturer´s protocol. Buffer exchange against PBS was performed using a HiTrap Desalting column (GE Healthcare).

### Affinity determination, receptor-ligand competition and simultaneous binding assay *via* biolayer interferometry

For affinity determination, anti-human IgG-Fc capture (AHC) biosensors were loaded with 10 µg/ml of the antibody of interest until a layer thickness of 1 nm was reached. For the following steps kinetics buffer (KB, Sartorius) was used. Association was measured for 300 s using varying concentrations of EGFR-ECD, PD-L1-ECD or CD16a-ECD (produced in-house) ranging from 7.8 nM to 500 nM followed by dissociation for 300 s. Binding kinetics were determined based on Savitzky-Golay filtering and a 1:1 Langmuir binding model.

For the EGF competition assay, AHC biosensors were loaded with 10 µg/ml of the antibody of interest until a layer thickness of 1 nm was reached. Subsequently, 250 nM EGFR-ECD pre-incubated with either 0 nM, 250 nM or 1000 nM monomeric EGF was applied for 600 s.

For the PD-1 competition assay, anti-human Fab-CH1 2nd Generation (FAB2G) biosensors were loaded with 10 µg/ml of the antibody of interest until a layer thickness of 1 nm was reached. Subsequently, 250 nM PD-L1-ECD pre-incubated with either 0 nM, 250 nM or 1000 nM PD-1-Fc was applied for 600 s.

For the simultaneous binding assay, anti-human IgG-Fc capture (AHC) biosensors were loaded with 2 µg of the KiH variant or the one-armed variant until a layer thickness of 1 nm was reached. After measuring association to 250 nM PD-L1-ECD for 200 s, 250 nM EGFR-ECD followed by 250 nM CD16a-ECD were added stepwise, each for a period of 200 s.

All measurements were performed using the Octet RED96 system (FortéBio, Molecular Devices) at 30°C and 1000 rpm.

### Thermal stability, SDS-PAGE and size exclusion chromatography

To determine thermal stability, antibodies were incubated with SYPRO Orange (Thermo Fisher Scientific) and a thermal shift assay was performed using a CFX Connect Real-Time PCR System (BioRad). The temperature gradient was set from 10°C to 95°C with an increment of 0.5°C/10s. The derivatives of the melting curves were determined using the CFX Maestro software to calculate the melting temperature (T_M_).

SDS-PAGE analysis was performed to characterize the produced antibodies. For this purpose, 3 µg of purified antibody were loaded onto a Mini-PROTEAN TGX 4-15% gel (BioRad) with either reducing or non-reducing Lämmli buffer and subsequently stained with Coomassie.

Size exclusion chromatography (SEC) using a Superdex 200 increase 10/300 GL column (Cytiva) together with an ÄKTA pure system (GE Healthcare) was performed at a flow rate of 0.15 mL/min for 20 min.

### Cultivation of A431, A549, MRC-5 and Daudi cells

A431 (DSMZ ACC 91), A549 (DSMZ ACC 107) and MRC-5 (ATCC CCL-171) cells were cultured in Dulbecco´s Eagle Medium (DMEM, Thermo Fisher), supplemented with 10% fetal bovine serum (FBS) superior (Merck Millipore) and 1x penicillin/streptomycin (Sigma Aldrich). Cells were cultured in T75 cell culture flasks at 37°C in a humidified atmosphere with 5% CO_2_ and passaged every 3-4 days after reaching 80% confluence. Daudi cells were maintained in RPMI 1640 supplemented with 20% FBS and 1x penicillin/streptomycin. Cells were subcultured every 2-3 days and incubated at 37°C and 5% CO_2._ Ready-to-use NHEK cells (PromoCell) were utilized for cellular binding studies.

### Cellular binding assay

Cellular binding of the produced antibodies was determined by affinity titration using EGFR/PD-L1 double positive A431 and A549 cells as well as EGFR positive MRC-5 and NHEK cells. To this end, 10^5^ cells/well were seeded in 96-well plates, washed with PBS-F [PBS + 2% (w/v) FBS] and subsequently incubated with the respective antibody in varying concentrations for 30 min on ice. Following another washing step, anti-human IgG-Fc PE-conjugated antibody was applied for 15 min. After washing, mean fluorescence was determined by flow cytometry using a CytoFLEX S (Beckman Coulter) and plotted against logarithmic antibody concentration. The resulting curves were fitted with a variable slope three-parameter fit using GraphPad Prism. All measurements were performed in duplicates, and the experiments were repeated at least three times, yielding comparable results. EGFR/PD-L1/CD16a triple negative Daudi cells were used to analyze nonspecific cellular binding.

### Antibody-dependent cell-mediated cytotoxicity (ADCC) reporter assay

The ADCC assay was performed using the Promega ADCC Reporter Bioassay Kit (G7010) according to the manufacturer’s protocol. The day before the assay, 10.000 A431 cells were seeded into a 96-well plate. A five-fold serial dilution of the antibodies of interest was tested (0.1 pM – 200 nM). The double Fab variant was analyzed in the range of 0.02 pM – 40nM. The assay was performed at 37°C and 5% CO_2_ for six hours. Luciferase activity was plotted against logarithmic antibody concentration and a variable slope four-parameter fit was used for fitting of the resulting curves.

## Data availability statement

The original contributions presented in the study are included in the article/**Supplementary Material**. Further inquiries can be directed to the corresponding author.

## Author contributions

JH and HK conceived and designed the majority of experiments. JH and SC performed the experiments. JH, SC, JB, KS, and HK analyzed the data. JG and BH gave scientific advice. JH and HK wrote the manuscript. All authors contributed to the article and approved the submitted version.
