# Supplementary material for: Generation of a symmetrical trispecific NK cell engager based on a two-in-one antibody
Source: Front Immunol. 2023 Apr 4;14:1170042. doi: 10.3389/fimmu.2023.1170042 (PMC10110854; doi:10.3389/fimmu.2023.1170042)
Supplement: Supplementary file 1 [file DataSheet_1.docx]

Supplementary Material

**Generation of a symmetrical trispecific NK cell engager based on a two-in-one antibody**

**Julia Harwardt, Stefania C. Carrara, Jan P. Bogen, Katrin Schoenfeld, Julius Grzeschik, Björn Hock and Harald Kolmar^*^**

***Correspondence:** Harald Kolmar: harald.kolmar@tu-darmstadt.de


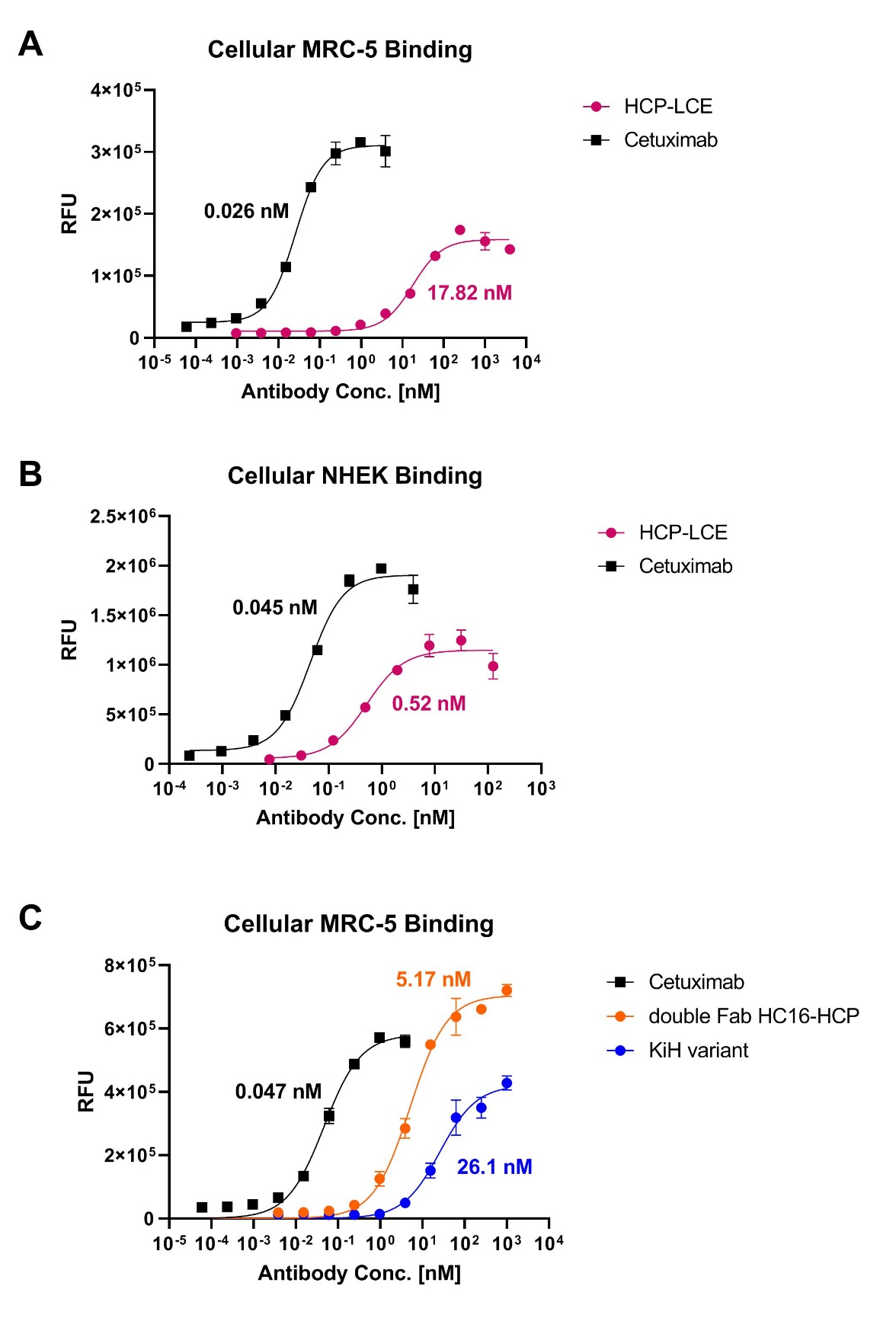


**Supplementary Figure 1.** Cellular binding on MRC-5 and NHEK cells. Cell titration of Cetuximab (black), HCP-LCE (pink), HC16-HCP (orange) and KiH variant (blue) on EGFR positive MRC-5 and NHEK cells. A variable slope three-parameter fit was utilized to for the resulting curves.


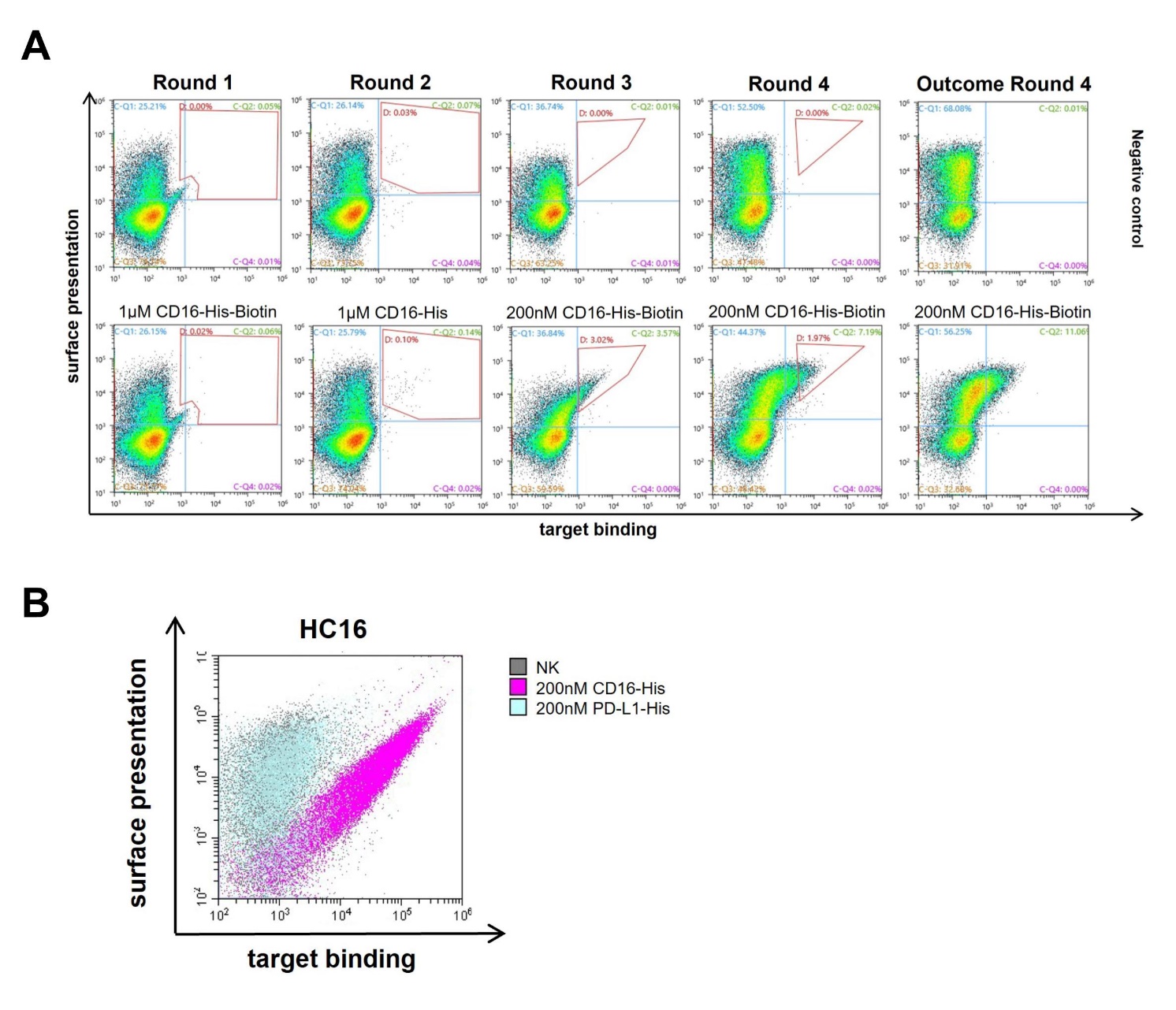


Supplementary Figure 2. Isolation of HC16. A) Sorting of the diploid common light chain yeast library. Surface presentation is depicted on the y-axis utilizing the anti-human lambda chain antibody PE labelled, while CD16-His-Biotin or CD16-His binding is shown on the x-axis using Streptavidin APC conjugated or the anti-6xHis AF647 antibody. B) Flow cytometric analysis of the isolated single clone after four consecutive rounds of FACS screening. Surface presentation is depicted on the y-axis utilizing the anti-human lambda chain antibody PE labelled, while CD16-His (pink) and PD-L1-His (blue) binding is shown on the x-axis using the anti-6xHis AF647 antibody.


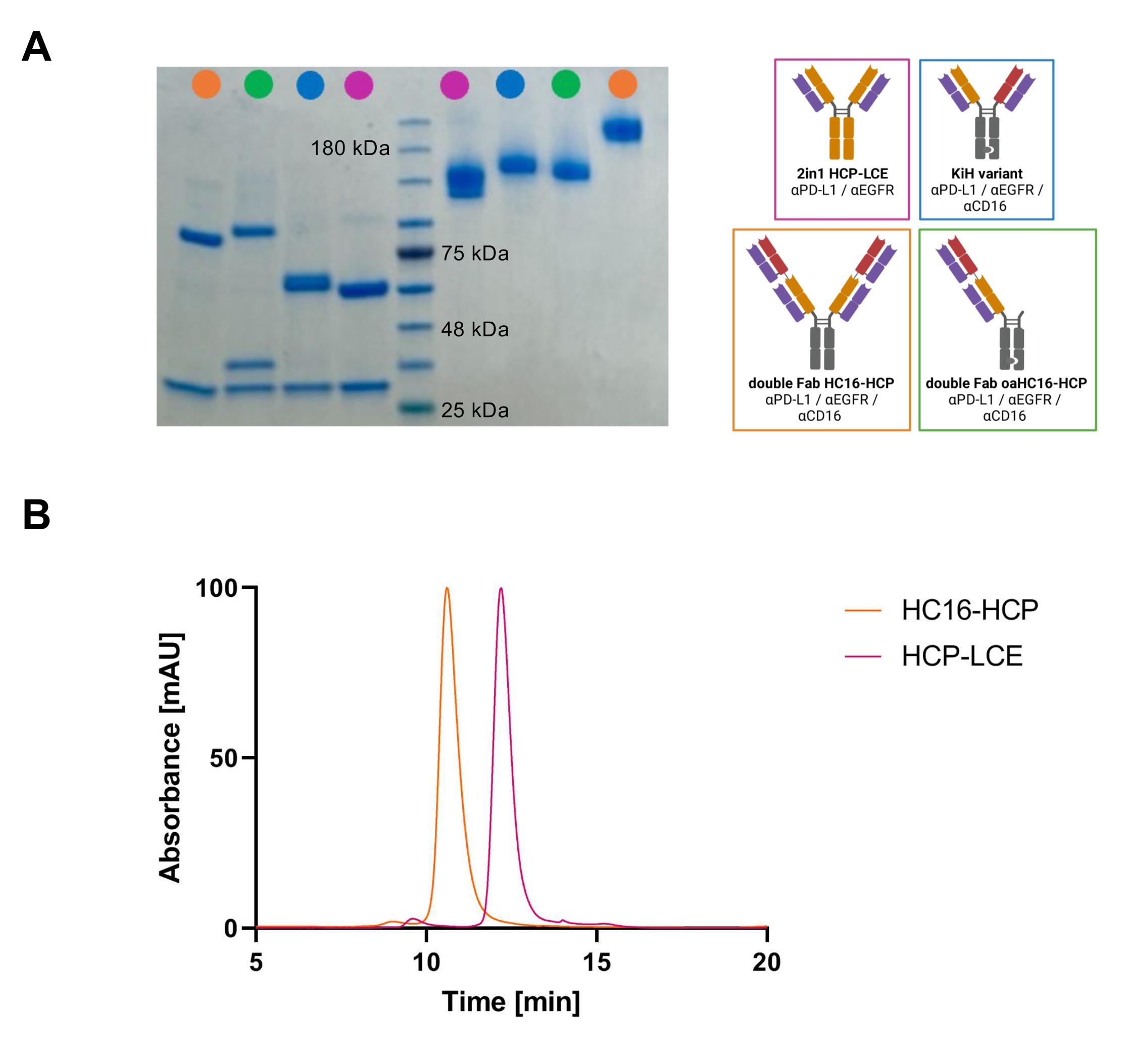


Supplementary Figure 3. SDS-PAGE analysis and SEC profiles. A) SDS-PAGE analysis of HC16-HCP (orange), oaHC16-HCP (green), KiH variant (blue) and HCP-LCE (pink) under reducing conditions (left) and under non-reducing conditions (right) revealing high purity and the expected molecular weights. B) SEC profiles of HCP-LCE (pink) and HC16-HCP (orange) revealing high purity and the expected molecular weights.


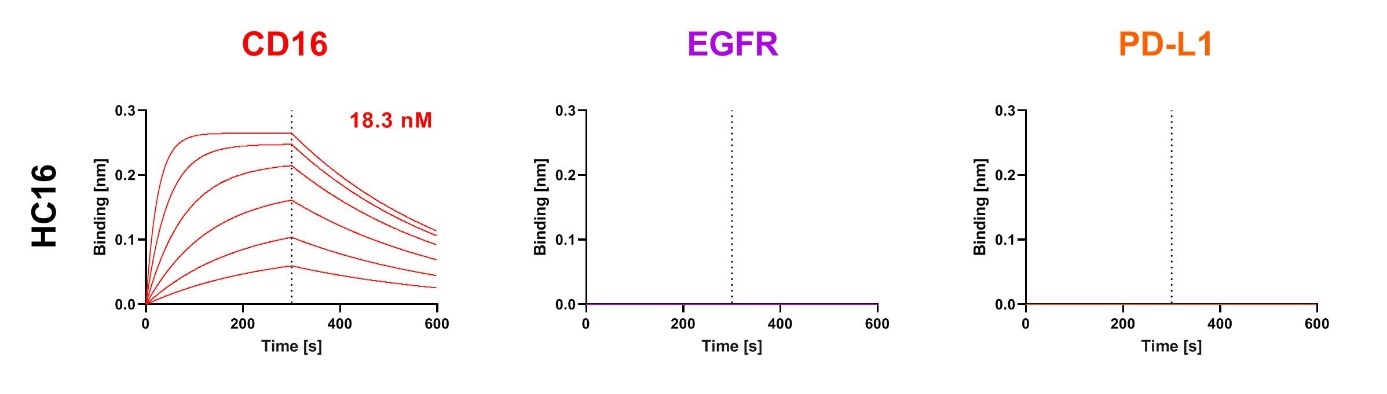


Supplementary Figure 4. Binding kinetics of HC16 by BLI-assisted measurements. BLI-measurement of the anti-CD16 antibody HC16 against CD16a, EGFR and PD-L1. The antibody targets CD16a exclusively.


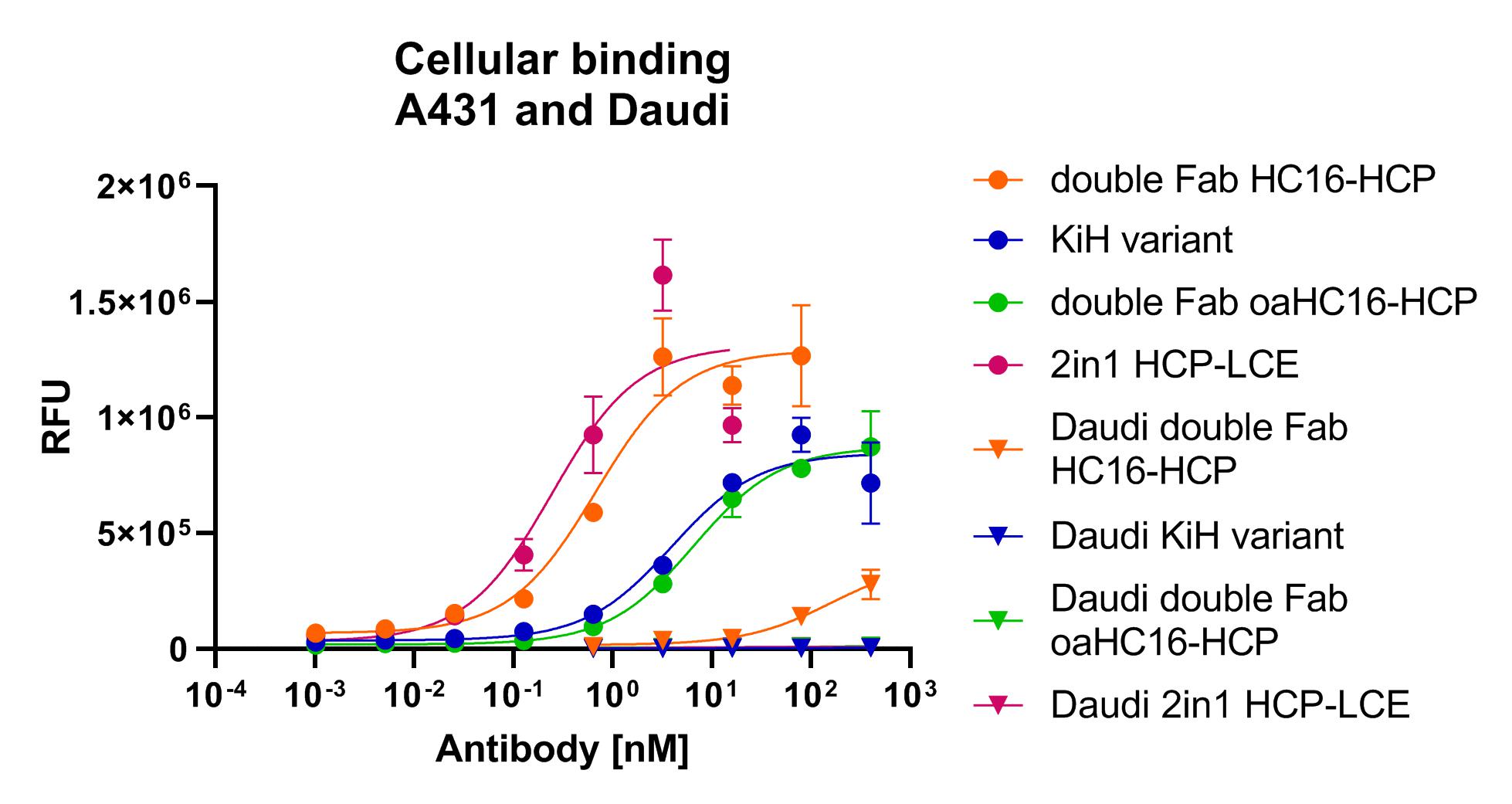


Supplementary Figure 5. Cellular binding on A431 cells and Daudi cells. Cell titration of HC16-HCP (orange), KiH variant (blue), oaHC16-HCP (green) and HCP-LCE (pink) on EGFR/PD-L1 double positive A431 cells (circles) and on EGFR/PD-L1 double negative Daudi cells (triangles). A variable slope three-parameter fit was utilized to fit the resulting curves. The assay was repeated twice, yielding comparable results.


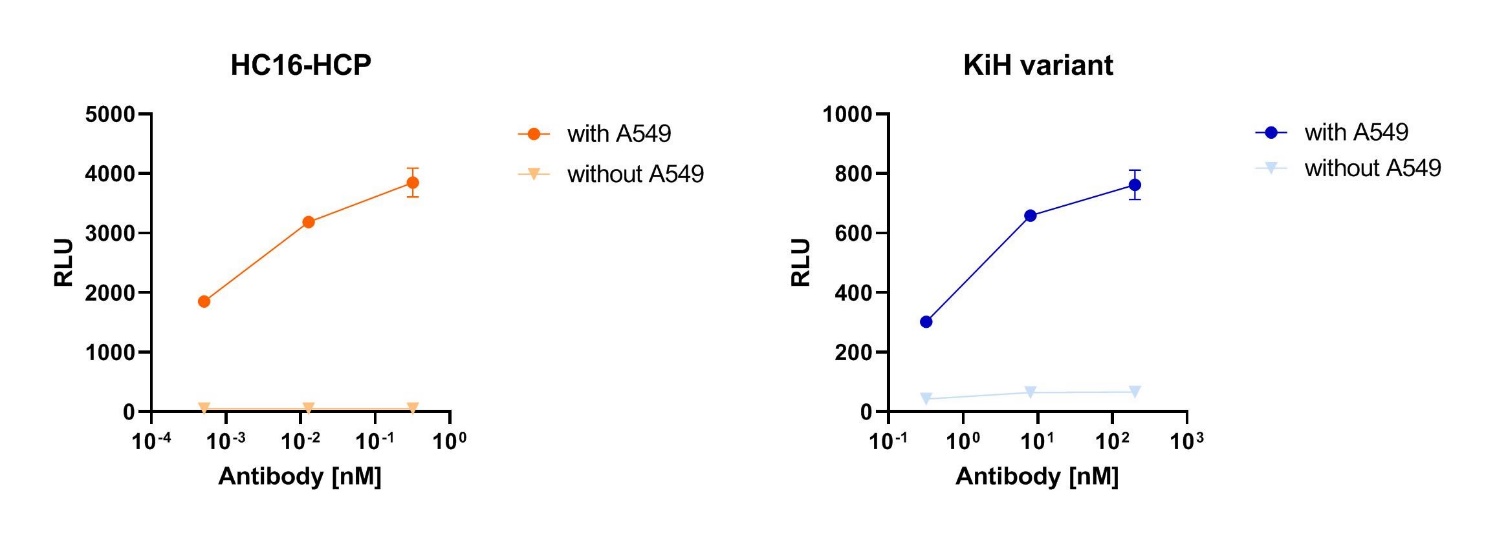

Supplementary Figure 6. ADCC cell-based reporter assay. The trispecific constructs HC16-HCP (orange) and KiH variant (blue) were tested in an ADCC reporter assay in the presence of EGFR/PD-L1 double-positive A549 cells (dots) and in the absence of A549 cells (triangles). Luciferase activity is plotted against the logarithmic antibody concentration. All measurements were performed in duplicates.
